# Single-center external validation and reconstruction of multiple predictive models for skip lateral lymph node metastasis in papillary thyroid carcinoma

**DOI:** 10.3389/fendo.2024.1366679

**Published:** 2024-08-23

**Authors:** Qi Li, Can Huang, Hongrui Zou, Jiaping Zhang, Jingwei Xin

**Affiliations:** ^1^ Department of Thyroid Surgery, China-Japan Union Hospital of Jilin University, Changchun, Jilin, China; ^2^ Department of Otolaryngology Surgery, China-Japan Union Hospital of Jilin University, Changchun, Jilin, China

**Keywords:** papillary thyroid carcinoma, skip lateral lymph node metastasis, external validation, nomogram, internal validation

## Abstract

**Objective:**

The unique metastatic pattern of skip lateral lymph node metastasis (SLLNM) in PTC patients may lead to missed diagnosis of lateral cervical metastatic lymph nodes. Therefore, many different SLLNM prediction models were constructed. In this study, partially eligible models (Hu 2020, Wang 2020, and Zhao 2023 nomograms) were selected for external validation, and then new variables were incorporated for model reconstruction to extend clinical applicability.

**Methods:**

576 PTC patients from our center were selected to evaluate the performance of the three nomograms using the receiver operating characteristic curve (ROC), calibration curves, and decision curve analyses (DCA). Three new variables were added to calibrate the model, including assessment of LN status on ultrasound (US-SLLNM), the distance from the tumor to the capsule (Capsular distance), and the number of central lymph node dissections (CLND number). Univariate and multivariate logistic regression analyses were used to screen independent predictors to reconstruct the model, and 1000 Bootstrap internal validations were performed.

**Results:**

SLLNM were present in 69/576 patients (12.0%). In external validation, the area under the ROC curves (AUCs) for Hu 2020, Wang 2020, and Zhao 2023 nomograms were 0.695 (95% CI:0.633-0.766), 0.792 (95% CI=0.73-0.845), and 0.769 (95% CI:0.713-0.824), respectively. The calibration curves for the three models were overall poorly fitted; DCA showed some net clinical benefit. Model differentiation and net clinical benefit improved by adding three new variables. Based on multivariate analysis, female, age, and maximum tumor diameter ≤ 10 mm, located at the upper pole, Capsular distance < 0mm, US-SLLNM, CLND number ≤ 5 were identified as independent predictors of SLLNM and were used to construct the new model. After 1000 Bootstrap internal validations, the mean AUC of the model was 0.870 (95% CI:0.839-0.901), the calibration curve was close to the ideal curve, and the net clinical benefit was significant.

**Conclusion:**

Overall, these nomograms were well differentiated and provided some net clinical benefit, but with varying degrees of underestimation or overestimation of the actual risk and high false-negative rates. New dynamic nomogram was constructed based on the addition of new variables and larger samples, showing better performance.

## 1 Introduction

Papillary thyroid carcinoma (PTC) is the most common thyroid malignancy, accounting for approximately 90% of all thyroid cancers (1). Generally, LNM of PTC occurs stepwise, first involving the central regional lymph nodes, then reaching the ipsilateral lateral cervical lymph nodes, and finally the contralateral lateral cervical and mediastinal compartments. However, some patients do not follow this pattern and develop lateral neck lymph node metastasis (LLNM) in the absence of central lymph node metastasis (CLNM), which is known as “skipping lateral lymph node metastasis (SLLNM)” (2, 3). This is not uncommon in clinical practice, and the frequency of skip metastases has been reported in the literature to range from 3.4% to 22.5% (2–17). Studies have shown that cervical lymph node metastasis increases the risk of local recurrence of PTC and decreases survival, especially in the lateral neck region (18–20). The prognosis of PTC patients with concomitant SLLNM was found to have a 10-year recurrence rate of 13% (20/151), which seriously affects patients’ quality of life (21).

However, prophylactic cervical lymph node dissection has been controversial. According to the 2015 version of the ATA guidelines and other studies, prophylactic central lymph node dissection is not recommended for patients with stage T1, T2, and noninvasive cN0 PTC; and prophylactic lateral lymph node dissection is not recommended if there is no preoperative evidence of lateral cervical lymph node metastasis as well as negative intraoperative central lymph nodes (22, 23). Ultrasound is one of the best methods for assessing the status of lymph nodes, but it is affected by complex anatomical structures, gas interference, and the subjective judgment of physicians, resulting in low sensitivity and high false-negative rates (24). Therefore, in-depth study of the pattern of SLLNM in patients with PTC and construction of relevant models are of great significance in guiding the treatment of patients, and it has been demonstrated that SLLNM in patients with PTC can be predicted (25).

At present, there are many constructed nomograms for predicting SLLNM. After rigorous screening (**Table 1**), three models of Hu 2020 (6), Wang 2020 (7) and Zhao 2023 (10) were selected. The original models showed good discrimination or consistency. Hu 2020, Wang 2020 were based on clinicopathological features, and Zhao 2023 was based on preoperative ultrasound features to construct prediction models, but the performance of the models was not externally verified. In this paper, a larger sample size will be used for external verification, and the evaluation of lymph node status under preoperative ultrasound (US-SLLNM) and the number of central lymph nodes dissected during operation (CLND number) will be used as one of the factors to explore. In addition, capsular invasion and ETE cannot be accurately judged under ultrasound. Therefore, the distance between the tumor and the capsule on ultrasound (Capsular distance) is used as another variable to explore new independent predictors, improve the predictive performance of the models, and provide help for the precise treatment of PTC patients.

**Table 1:** Relevant information on the constructed models for predicting SLLNM.

Study (First author)	Country	Data time	Inclusion Criteria	Skip (n)	Total (n)	Skip rate	grouping criteria	Model factors	Modeling methods	
Hu D (6), 2020 May	China	2012.1 │ 2017.12	Total thyroidectomy CLND LLND	72	745	9.7%	CLNM LLNM VS No CLNM LLNM	Age (>55) Unilateral Location (upper pole)	Nomogram
Wang W (7), 2020 Jul	China	2018.3 │ 2019.7	Total thyroidectomy CLND LLND	44	378	11.6%	CLNM LLNM VS No CLNM LLNM	Size (≤ 1cm) Location (upper pole) Age (larger)	Nomogram
Zhu S (8), 2022 Jul	China	2016.1 │ 2019.9	Thyroidectomy CLND LLND	106	819	12.9%	CLNM LLNM VS No CLNM LLNM	Size (≤ 1cm) ETE HT Location (upper pole) CLND number (less) BRAF V600E mutation	SVM
Yang Z (9), 2021 Sep	China	2017.6 │ 2019.6	Total thyroidectomy CLND LLND (With/without)	37	1075	3.4%	No CLNM No LLNM VS No CLNM LLNM	Capsular invasion Size (≥1cm) Multifocality Location (upper pole)	Nomogram
Zhao M (10), 2023 May	China	2019.1 │ 2021.12	Total thyroidectomy CLND LLND	41	267	15.4%	CLNM LLNM VS No CLNM LLNM	Age (>40) US-Size (<0.91cm) Location (upper pole) Margin (Non-smooth) ETE	Nomogram
Jiwang L (17), 2023 Sep	China	2016 │ 2020	Thyroidectomy CLND LLND (With/without)	111	1037	10.7%	Other types VS No CLNM LLNM	Sex (Female) Location ETE calcification	Nomogram

SVM, support vector machine; ETE, Extraglandular expansion; HT, Hashimoto’s thyroiditis; NG, Nodular goiter.

## 2 Materials and methods

### 2.1 Research subjects

This study used a single-center retrospective analysis. Approval was obtained from the Research Ethics Committee (2023072602), and the requirement for informed consent was waived.

A total of 576 patients from the China-Japan Union Hospital of Jilin University from January 2020 to December 2022 were retrieved from the electronic medical record system. Inclusion criteria: (1) postoperative pathology confirmed as PTC (including papillary carcinoma, papillary adenocarcinoma, papillary carcinoma follicular subtype, micropapillary carcinoma); (2) First thyroid surgery with total thyroidectomy, central lymph node dissection and lateral cervical lymph node dissection; (3) Postoperative pathology confirmed the presence of LLNM; (4) No history of head and neck radiation exposure.

Exclusion criteria: (1) other types of thyroid cancer (including follicular thyroid cancer, medullary thyroid cancer, undifferentiated thyroid cancer, and high-risk subtypes of PTC such as diffuse sclerosis); (2) previous history of thyroid surgery; (3) PTC patients with tumors located in the isthmus or occupying most or even all of the glands (to better determine the effect of tumor location on SLLNM); (4) Postoperative pathology confirmed that there was no LLNM; (5) There were other malignant tumors; (6) History of head and neck radiotherapy.

### 2.2 Surgery treatment

In this study, all patients underwent total thyroidectomy, central lymph node dissection, and lateral cervical lymph node dissection. Central lymph node dissection ranged from superiorly by the hyoid bone, inferiorly to the sternal notch, and lateral to the carotid sheath, posteriorly to prevertebral fascia. Lateral lymph node dissection covers zones II, III, IV, and V. Zone I or VII dissection was performed only if evidence of metastasis was found on imaging, cytopathology, or intraoperatively. In our study, no patient underwent zone I and VII clearance. All enrolled patients had preoperative findings of suspected LLNM or were at high risk of LLNM based on clinician experience.

### 2.3 Data collection

Three nomograms used to predict SLLNM were considered in our study. For all included patients, parameters related to Hu 2020 nomogram were collected, such as age (> 55, ≤ 55), bilaterality under pathology, and location (upper pole, non-upper pole); Wang 2020 nomogram parameters such as age, pathological tumor size (≤ 10mm, > 10mm), location (upper, middle and lower poles); Zhao 2023 nomogram parameters such as age (> 40, ≤ 40), tumor size under ultrasound (< 9.1 mm, ≥ 9.1 mm), location (upper pole, non-upper pole), margin and extrathyroidal extension (ETE), as shown in **Table 1**. In addition to this, patient’s gender, BMI; ultrasound characteristics such as bilaterality, multifocality, calcification status, aspect ratio, and nodule blood supply; and pathologic characteristics such as bilaterality, multifocality, capsular invasion, ETE, Hashimoto’s thyroiditis (HT), nodular goiter (NG). In the above three models, the grouping criteria for some variables were inconsistent; for example, for multifocal, Hu 2020 specified ≥2 lesions in a unilateral lobe, and Wang 2020 and Zhao 2023 specified ≥2 PTC lesions in bilateral lobes; the grouping criteria for age, location, and size were also inconsistent. Here, capsular invasion refers to the tumor invading the thyroid capsule but not penetrating it. ETE is the tumor penetrating the capsule or invading the surrounding soft tissues and structures (26).

**Figure 1:**
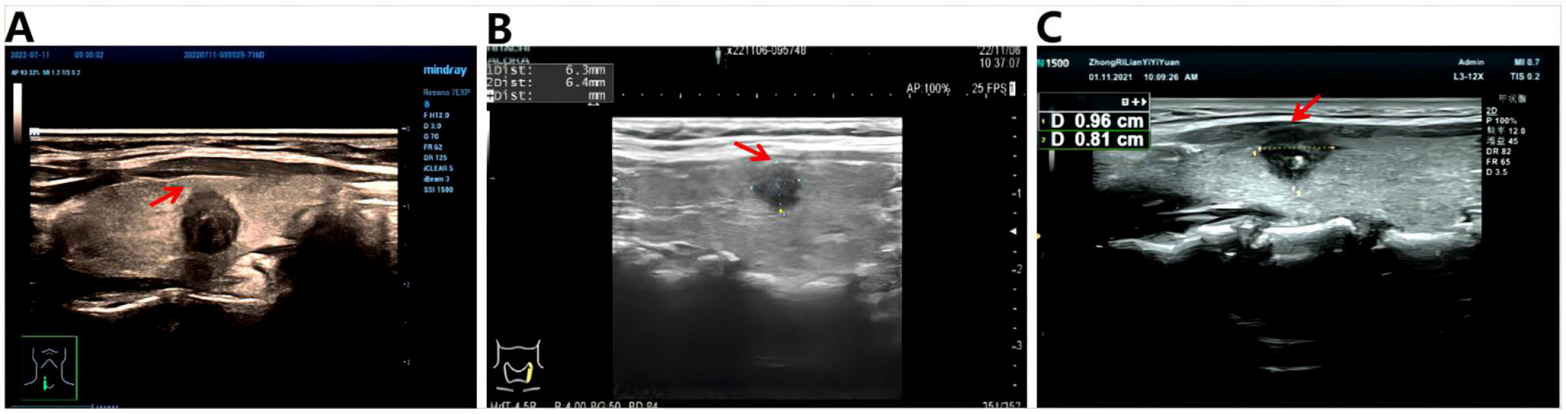
Schematic diagram of capsule distance. **(A)** >0mm, indicating that the tumor is completely within the gland; **(B)** =0mm, indicating that the tumor is close to the capsule; **(C)** <0mm, which means the tumor breaks through the capsule.

In addition, three variables, Capsular distance (**Figure 1**), US-SLNM, and intraoperative CLND number, were collected into the model to see whether there was a gain. US-SLLNM, referring to the 2013 European Thyroid Association guidelines (27), which divided lymph nodes into normal, uncertain, and suspected malignant; uncertain and suspicious malignancy on ultrasound was defined as suspicious lymph nodes; no abnormality was found in the central lymph nodes under ultrasound, and the suspicious cases of lateral cervical lymph nodes were positive.

Hu 2020, Wang 2020 and Zhao 2023 nomograms are shown in the **Supplementary Figures**.

### 2.4 statistical analysis

**Figure 2:**
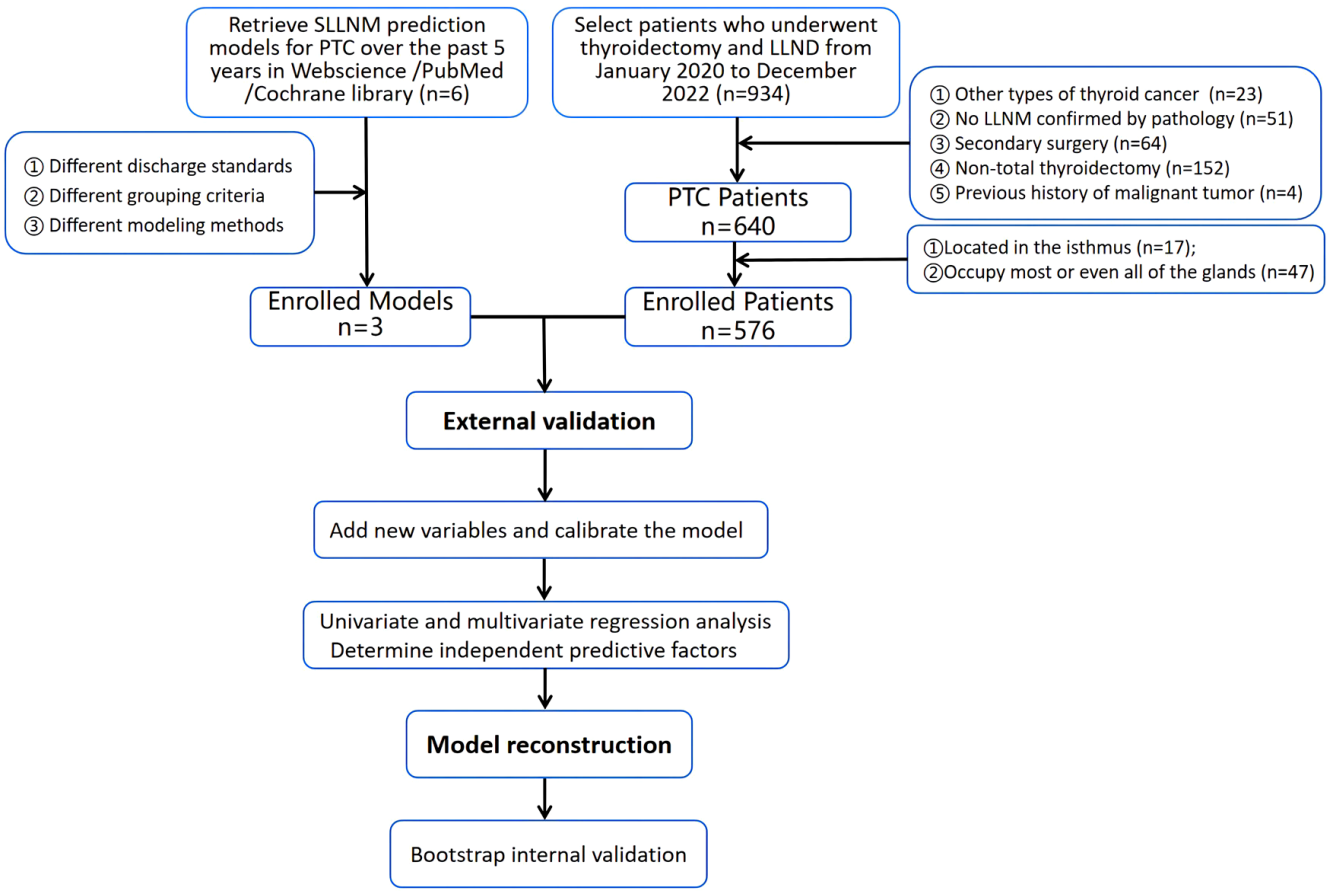
Flow chart of the entire experiment.

Statistical analysis was performed using R software 4.3.0 and SPSS 26.0 software. Categorical variables were described as frequencies and percentages; continuous variables were expressed as means and standard deviations. The predictive performance of original nomograms was assessed by area under the ROC curve (AUC), calibration curves, and decision curve analysis (DCA). AUC values quantified the differentiation of the external validation cohort across the three nomograms; calibration curves were used to assess the agreement between predicted and actual SLLNM risk; and DCA was used to visualize the net benefit of the three nomograms. Taking SLLNM or not as the dependent variable, the predicted probability of the original nomogram and Capsular distance, US-SLLNM, and CLND number as the independent variables, logistic regression was used to incorporate each of the three new variables into the original nomograms and to plot the ROC curves and the DCA in order to compare the predictive performance of the original model to the model with the addition of the new variables. Next, univariate and multivariate logistic regression analyses were performed based on larger sample sizes to screen for stable independent predictors and construct a dynamic nomogram, with values of *P*<0.05 considered statistically significantly different. Internal validation was performed using 1000 Bootstrap, ROC curves, and calibration curves were used to evaluate the predictive accuracy and consistency of the model, and DCA reflected the net benefit of the model to patients. The original models were all static nomograms, and the regression parameters of the models were not provided, so we used an approximation, which is also the only approach used clinically. We placed the information of each patient into three original models to obtain the corresponding total score and predicted probability. Flow chart of the entire experiment is shown in **Figure 2**.

## 3 Results

### 3.1 Baseline characteristics

In the external cohort, the incidence of SLLNM was 12.0%, while the incidence of SLLNM was 9.7%, 11.6%, and 15.4% in Hu 2020 (Model 1), Wang 2020 (Model 2), and Zhao 2023 (Model 3), respectively. The baseline characteristics of the external validation cohort and the original cohort of the three models are shown in **Table 2**.

**Table 2:** Comparison of baseline characteristics.

Factors	External verification	Model 1	Model 2	Model 3
	N=576	N=745	N=378	N=267
Sex				
Female	391(67.9%)	506 (67.9%)	264 (69.84%)	163(61.1%)
Male	185(32.1%)	239 (32.1%)	114 (30.16%)	104(38.9%)
Age	40.15± 10.604	41.1 ± 13.7	39.96 ± 11.73	35.03 ± 11.318
Age1				
<55	513(89.1%)		336 (88.89%)	233(87.3%)
≥55	63(10.9%)		42 (11.11%)	34(12.7%)
Age2				
≤55	522(90.6%)	480 (64.4%)		
>55	54(9.4%)	265 (35.6%)		
BMI				
<25	296(51.4%)			138(51.7%)
≥25	280(48.6%)			129(48.3%)
US-Maximum diameter	1.505 ± 0.723			1.506 ± 0.721
Tumor location 1				
Upper	198(34.4%)		92 (24.34%)	
Middle	267(46.4%)		180 (47.62%)	
Lower	111(19.3%)		106 (28.04%)	
Tumor location 2				
Upper	198(34.4%)	226 (30.3%)		102(38.2%)
Un-upper	378(65.6%)	519(69.7%)		165(61.8%)
US-Multifocality				
Absent	219(38.0%)			156(58.4%)
Present	357(62.0%)			111(41.6%)
US-Bilaterality				
Absent	306(53.1%)			199(74.5%)
Present	270(46.9%)			68(25.5%)
US-ETE				
Absent	518(89.9%)			210(78.7%)
Present	58(10.1%)			57(21.3%)
US-Margin				
Smooth	44(7.6%)			160(59.9%)
Non-smooth	532(92.4%)			107(40.1%)
Maximum diameter				
≤10mm	249(43.2%)	183 (24.6%)	142 (37.57%)	91(34.1%)
>10mm	327(56.8%)	562 (75.4%)	236 (62.43%)	176(65.9%)
Multifocality 1				
Absent	212(36.8%)		294 (77.78%)	104(39.0%)
Present	364(63.2%)		84 (22.22%)	163(61.0%)
Multifocality 2				
Absent	339(58.9%)	635 (85.2%)		
Present	237(41.1%)	110 (14.8%)		
Bilaterality				
Absent	276(47.9%)	573 (76.9%)	261 (69.05%)	142(53.2%)
Present	300(52.1%)	172 (23.1%)	117 (30.95%)	125(46.8%)
Capsular invasion				
Absent	508(88.2%)		283(74.87%)	
Present	68(11.8%)		95 (25.13%)	
ETE				
Absent	454(78.8%)	539 (72.3%)	309(81.75%)	195(73.0%)
Present	122(21.2%)	206 (27.7%)	69(18.25%)	72(27.0%)
HT				
Absent	472(81.9%)	646 (86.7%)	291 (76.98%)	185(69.3%)
Present	104(18.1%)	99 (13.3%)	87 (23.16%)	82(30.7%)
NG				
Absent	249(43.2%)			205(76.8%)
Present	327(56.8%)			62(23.2%)
CLND number	9.01 ± 5.347	11.1 ± 6.5	7.51 ± 5.08	8.81 ± 5.684
LLND number	23.66 ± 11.772	21.2 ± 12.3	18.25 ± 12.59	24.19 ± 13.354
LLNM number	4.99 ± 3.674	4.2 ± 4.4	–	5.48 ± 4.137

HT, Hashimoto’s thyroiditis; NG, Nodular goiter; US-Maximum diameter, US-Multifocality, US-Bilaterality, US-ETE and US-Margin refer to the tumor characteristics under ultrasound conditions. Multifocality 1,This refers to double lobes >2 cancer foci. Multifocality 2, This is defined as >2 cancer lesions in a single lobe. For tumor characterization in multifocal patients, the most suspicious or largest is selected as the dominant nodule.

### 3.2 Risk assessment of the original nomograms

**Figure 3:**
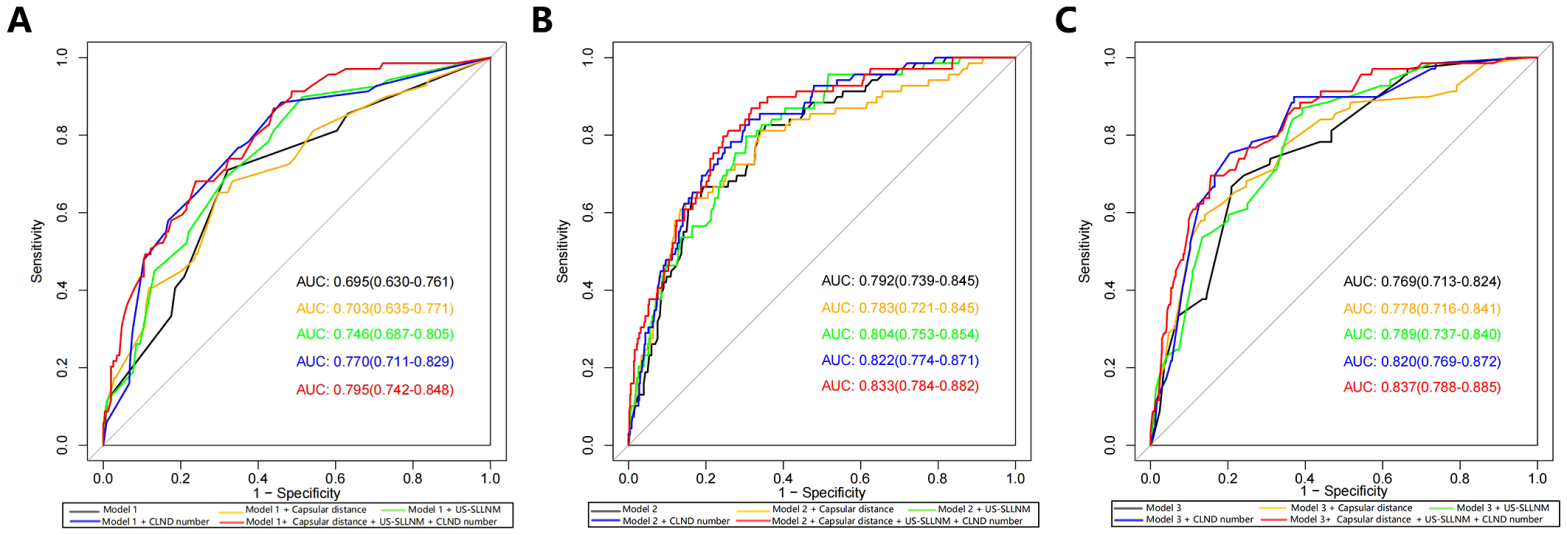
ROC curves **(A)** Model 1; **(B)** Model 2; **(C)** Model 3. AUCs from top to bottom are original model, model with added Capsular distance, model with added US-SLLNM, model with added CLND number, model with added three factors. ROC, receiver operating characteristic curve; AUC, Area under the curve; CLND, Central lymph node dissection.

None of the original models provided the optimal cutoff value; therefore, in the external validation queue, the cutoff value when maximizing the Yoden index was selected based on the ROC curves of each model (**Figure 3**). From the ROC curves of the three models, it is easy to see that, although they are all reduced to a certain extent compared with the original models, they also all show a better degree of differentiation, see **Tables 3**, **4**.

**Table 3:** Risk assessment of original models.

	Model 1		Model 2		Model 3		Actual situation
	Predict	Actual	Predict	Actual	Predict	Actual	
AUC (95CI%)	0.695(0.630-0.761)		0.792(0.739-0.845)		0.769(0.713-0.824)		-
Sensitivity	71.0%		63.8%		66.7%		-
Specificity	67.9%		83.6%		79.1%		-
Cut-point	0.082	-	0.264	-	0.317	-	-
SLLNM (+)	212	49	127	44	158	46	69
SLLNM (-)	364	344	449	424	418	395	507

**Table 4:** Comparison of ROC curves for original, external, and corrected cohorts.

	Original queue ROC curve			External queue ROC curve			Corrected queue ROC curve		
	AUC 95%CI	sensitivity	specificity	AUC 95%CI	sensitivity	specificity	AUC 95%CI	sensitivity	specificity
Model 1	0.734 (0.671–0.796)	71.1%	68.5%	0.695 (0.630-0.761)	71.0%	67.9%	0.795 (0.742-0.848)	68.1%	76.1%
Model 2	0.806 (0.736–0.876)	79.5%	67.7%	0.792 (0.739-0.845)	63.8%	83.6%	0.833 (0.784-0.882)	81.2%	74.2%
Model 3	0.797 (0.726-0.867)	90.2%	50.3%	0.769 (0.713-0.824)	66.7%	79.1%	0.837 (0.788-0.885)	69.6%	84.4%

ROC, receiver operating characteristic curve; AUC, area under the curve.

Model 1: The optimal cutoff value for this nomogram was 8.2% (AUC = 0.695, 95CI%: 0.630-0.761), and sensitivity and specificity associated with the optimal threshold were 71.0% and 67.9%, respectively. Applying this cutoff value, 29.0% (20/69) of SLLNM-positive patients would be below the threshold, would be incorrectly judged to be at low risk of developing SLLNM, and would be advised against the need for lateral cervical lymph node dissection.

Model 2: The optimal cutoff value of this nomogram was 26.4% (AUC = 0.792, 95CI%: 0.739-0.845), and sensitivity and specificity associated with the optimal threshold were 63.8% and 83.6%, respectively. Applying this cutoff value, 36.2% (25/69) of patients would have escaped positive screening.

Model 3: The optimal cutoff value for this nomogram was 31.7% (AUC = 0.769, 95CI%: 0.713-0.824), and sensitivity and specificity associated with the optimal threshold were 66.7%, respectively, 79.1%. Applying this cutoff value, 33.3% (23/69) of patients would miss positive screening. We believe that this non-negligible percentage of patients is too high and that this finding represents a major limitation of the original nomograms.

### 3.3 Performance of the original nomograms

**Figure 4:**
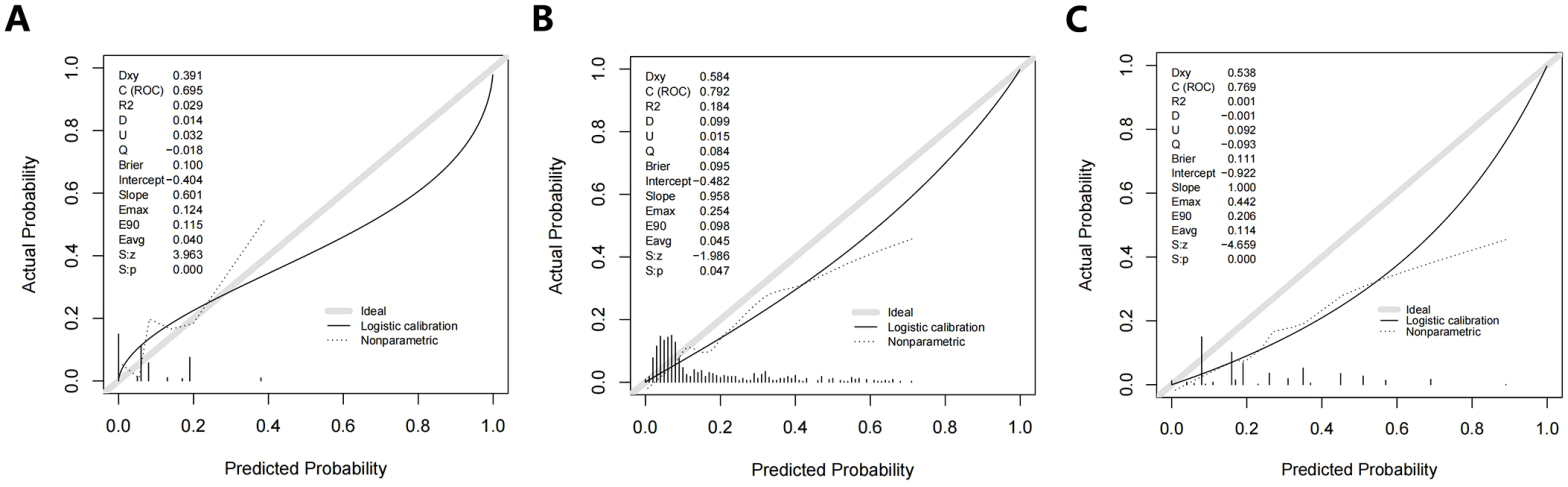
Calibration curves **(A)** Model 1; **(B)** Model 2; **(C)** Model 3.

The calibration curves for Model 1, Model 2, and Model 3 are shown in **Figure 4**. Overall, the three original models passed the Hosmer-Lemeshow goodness-of-fit test, which showed that the models all fit poorly (P < 0.05.) Model 1 was more consistent at predicted probabilities of 15.5% to 24.5% and underestimated the risk of SLLNM in the current cohort at predicted probabilities of 6.5% to 15.5% and >24.5% (**Figure 4A**). With Model 2, there is more agreement with actual risk at predicted probabilities <35%; however, when larger predicted probabilities (>35%) occur, there tends to be an overestimation of observed risk (**Figure 4B**). Model 3, on the other hand, has a degree of overestimation over the entire range of predicted probabilities (**Figure 4C**). The current incidence of SLLNM ranges from 3.4% to 22.5%, as described in the relevant literature (2–17). Thus, in the lower-risk patient cohort, Model 1 underestimates the actual risk to some extent, Model 2 is more consistent with the actual risk, and Model 3 overestimates the actual risk.

**Figure 5:**
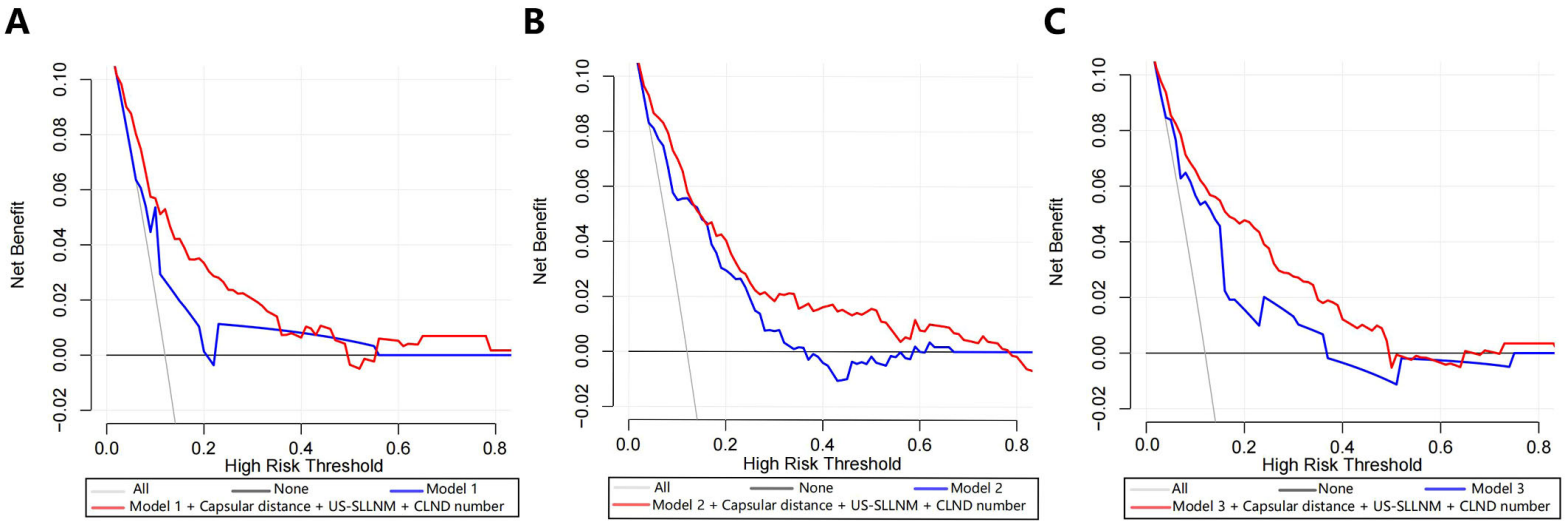
Decision curve analyses (including original model, model with added three factors). **(A)** Model 1; **(B)** Model 2; **(C)** Model 3.

The DCA of Model 1 showed that the threshold probability was between 6.5% -20% and 22% -56%, with a certain clinical net benefit. The optimal Cutoff value of this nomogram is 8.2%, and the use of which will bring a net clinical benefit of 5.5% (**Figure 5A**). The DCA of Model 2 and Mode 3 showed a net clinical benefit when the threshold probabilities were 17.9% -36.3% and 4.1% -37%, respectively (**Figures 5B, C**). When the respective optimal Cutoff values are taken, there is also a certain clinical net benefit, indicating that the use of this nomogram has clinical significance.

### 3.4 Performance of nomograms after adding new variables

**Figure 6:**
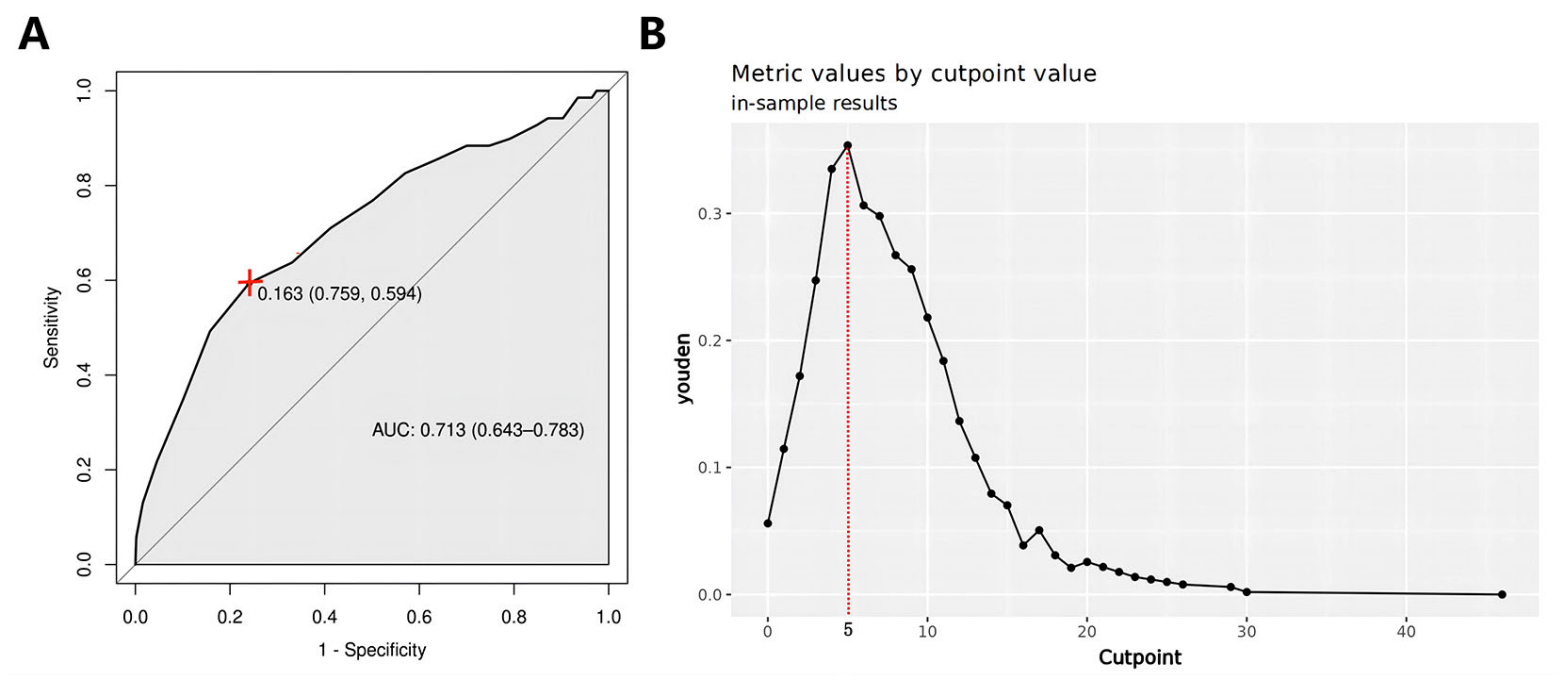
**(A)** CLND number ROC curve; **(B)** CLND number Optimal cut-point plot. ROC, receiver operating characteristic curve; CLND, central lymph node dissections.

Three new variables, Capsular distance, US-SLLNM, and CLND number, were included in this study. The ROC curve analysis of the CLND number showed that the AUC was 0.713 (0.643-0.783) (**Figure 6A**). In order to divide its optimal cut-point, this study selected the cutoff when the Yoden index is at its maximum, which is 5 (**Figure 6B**). As can be seen from **Figure 3**, except for the Capsular distance in Model 2, which did not increase the AUC (**Figure 3B**), the remaining two variables increased the AUC to varying degrees in all three original models. Comparison of AUCs for the original cohort, the external cohort, and the cohort calibrated with the added variables is shown in **Table 4**. The DCA in **Figure 5** shows that the model with the addition of the three variables resulted in a significant increase in both the risk threshold range and the net benefit compared to the original model.

## 4 Model construction

### 4.1 Risk factors for cervical SLLNM in PTC patients

Based on the gain brought by the new variables to the model, a larger sample size (576 cases) will be used this time to explore the influences of previous studies again, in order to construct a model with higher performance. The sample sizes for the three nomograms constructed above were 521, 378, and 267, respectively. In the univariate logistic regression analysis, it was found that female (*P*=0.014); age (*P*<0.001); BMI ≥25Kg/m^2^ (*P*=0.030); upper pole (*P*<0.001); maximal diameter of the tumor ≤10mm (*P*=0.009); Capsular distance < 0mm (*P*=0.006); US-SLLNM (*P*<0.001); and CLND number ≤ 5 (*P*<0.001) were significantly associated with SLLNM in PTC patients (*P*<0.05). On the contrary, the differences in margins, aspect ratio, nodule blood supply, calcification, bilaterality, multifocality, capsular invasion, ETE, HT, and NG were not statistically significant between the SLLNM (+) group and the SLLNM (-) group (all *P*>0.05). The results of univariate logistic regression analysis are shown in **Table 5**.

**Table 5:** Univariate Logistic regression analysis of risk factors for SLLNM in PTC patients.

Factors	SLLNM (-)	SLLNM (+)	OR	95%CI	*P*-value
	N=507	N=69			
Sex, n (%)					
Male	172 (33.9%)	13 (18.8%)	1.000 (Reference)		
Female	335 (66.1%)	56 (81.2%)	2.212	1.177,4.156	0.014
**Age (mean ± SD, years)**	39.15 ± 10.208	47.51 ± 10.628	1.077	1.051,1.104	<0.001
BMI, n (%)					
<25	252 (49.7%)	44 (63.8%)	1.000 (Reference)		
≥25	255 (50.3%)	25 (36.2%)	0.561	0.334,0.945	0.030
^1^Margin, n (%)					
Smooth	37 (7.3%)	7 (10.1%)	1.000 (Reference)		
Non-smooth	470 (92.7%)	62 (89.9%)	0.697	0.298,1.632	0.406
^1^Aspect ratio, n (%)					
<1	270 (53.3%)	31 (44.9%)	1.000 (Reference)		
>1	237 (46.7%)	38 (55.1%)	1.396	0.842,2.315	0.195
^1^Nodule Blood Supply, n (%)					
No	396 (78.1%)	55 (79.7%)	1.000 (Reference)		
Little	79 (15.6%)	8 (11.6%)	0.729	0.334,1.590	0.427
Rich	32 (6.3%)	6 (8.7%)	1.350	0.540,3.375	0.521
^1^Calcification, n (%)					
Absent	93 (18.3%)	17 (24.6%)	1.000 (Reference)		
Microcalcification	324 (63.9%)	42 (60.9%)	0.709	0.386,1.304	0.268
Macrocalcification	90 (17.8%)	10 (14.5%)	0.608	0.264,1.398	0.242
^1^Tumor location1, n (%)					
Middle	251 (49.5%)	16 (23.2%)	1.000 (Reference)		
Lower	105 (20.7%)	6 (8.7%)	0.896	0.341,2.354	0.824
Upper	151 (29.8%)	47 (68.1%)	4.883	2.674,8.916	<0.001
^2^Maximum diameter, n (%)					
>10mm	298 (58.8%)	29 (42.0%)	1.000 (Reference)		
≤10mm	209 (41.2%)	40 (58.0%)	1.967	1.181,3.274	0.009
^2^Multifocality1, n (%)					
Absent	183 (36.1%)	29 (42.0%)	1.000 (Reference)		
Present	324 (63.9%)	40 (58.0%)	0.779	0.467,1.299	0.338
^2^Bilaterality, n (%)					
Absent	244 (48.1%)	32 (46.4%)	1.000 (Reference)		
Present	263 (51.9%)	37 (53.6%)	1.073	0.648,1.776	0.785
^2^Capsular invasion, n (%)					
Absent	449 (88.6%)	59 (85.5%)	1.000 (Reference)		
Present	58 (11.4%)	10 (14.5%)	1.312	0.636,2.706	0.462
^2^ETE, n (%)					
Absent	400 (78.9%)	54 (78.3%)	1.000 (Reference)		
Present	107 (21.1%)	15 (21.7%)	1.038	0.564,1.912	0.904
^2^HT, n (%)					
Absent	420 (82.8%)	52 (75.4%)	1.000 (Reference)		
Present	87 (17.2%)	17 (24.6%)	1.578	0.871,2.859	0.132
^2^NG, n (%)					
Absent	215 (42.4%)	34 (49.3%)	1.000 (Reference)		
Present	292 (57.6%)	35 (50.7%)	0.758	0.458,1.254	0.281
^1^Capsular distance, n (%)					
>0mm	209 (41.2%)	18 (26.1%)	1.000 (Reference)		
=0mm	252 (49.7%)	39 (56.5%)	1.797	0.998,3.235	0.051
<0mm	46 (9.1%)	12 (17.4%)	3.029	1.365,6.722	0.006
^1^US-SLLNM, n (%)					
No	346 (68.2%)	28 (40.6%)	1.000 (Reference)		
Yes	161 (31.8%)	41 (59.4%)	3.147	1.879,5.270	<0.001
CLND number, n (%)					
**>5**	385 (75.9%)	28 (40.6%)	1.000 (Reference)		
**≤5**	122 (24.1%)	41 (59.4%)	4.621	2.742,7.787	<0.001

^1^Tumor characteristics under ultrasound condition; ^2^Tumor characteristics in pathological state.

Multifocality: this refers to double lobes >2 cancer foci.

### 4.2 Independent risk factors for cervical SLLNM in PTC patients

The eight variables that were statistically different from those mentioned above were included in the multivariate logistic regression, and the results showed that female (OR 2.740, 95%CI 1.279-5.870, *P*=0.009), age (OR 1.061, 95%CI 1.031-1.091, *P*<0.001), tumor located in the upper pole (OR 3.427, 95%CI 1.749- 6.715, *P* < 0.001), maximum tumor diameter ≤ 10 mm (OR 2.544, 95%CI 1.360-4.758, *P* = 0.003), Capsular distance < 0 mm (OR 3.287, 95%CI 1.217-8.875, *P* = 0.019), US-SLLNM (OR 3.009, 95%CI 1.635-5.536, *P*<0.001), and CLND number ≤5 (OR 3.244, 95%CI 1.766-5.959, *P*<0.001) were independent risk factors for cervical SLLNM in PTC patients. The results of the multivariate logistic regression analysis are shown in **Table 6**.

**Table 6:** Multivariate logistic regression analysis of risk factors for SLLNM in PTC patients.

Factors	OR	95%CI	*P*-value
χ_1_Sex (Female)	2.740	1.279,5.870	0.009
χ_2_Age	1.061	1.031, 1.091	<0.001
χ_3_BMI (≥25)	0.700	0.369,1.326	0.274
χ_4_Tumor location			
Middle	—	—	
Lower	0.749	0.266, 2.107	0.583
Upper	3.427	1.749, 6.715	<0.001
χ_5_Maximum diameter (≤10mm)	2.544	1.360, 4.758	0.003
χ_6_Capsular distance			
>0mm	—	—	
=0mm	1.832	0.935, 3.587	0.078
<0mm	3.287	1.217, 8.875	0.019
χ_7_US-SLLNM (Yes)	3.009	1.635, 5.536	<0.001
χ_8_CLND number (≤5)	3.244	1.766, 5.959	<0.001

### 4.3 Construction and validation of the nomogram

Based on the seven independent risk factors described above, the logistic regression equation of the resulting model is as follows: Y= -7.893 + 1.119(χ_1_)+ 0.060(χ_2_) - 0.298(χ_4-2_) + 1.239(χ_4-3_) + 0.978(χ_5_) + 0.613(χ_6-2_) + 1.203(χ_6-3_) + 1.053(χ_7_) + 1.173(χ_8_) (χ_1_:Female; χ_2_:Age value; χ_4-2_: Lower; χ_4-3_:Upper; χ_5_:Maximum diameter ≤ 10mm; χ_6-2_:Capsular distance=0mm; χ_6-3_:Capsular distance<0mm; χ_7_:US-SLLNM(Yes); χ_8_:CLND number ≤ 5).

**Figure 7:**
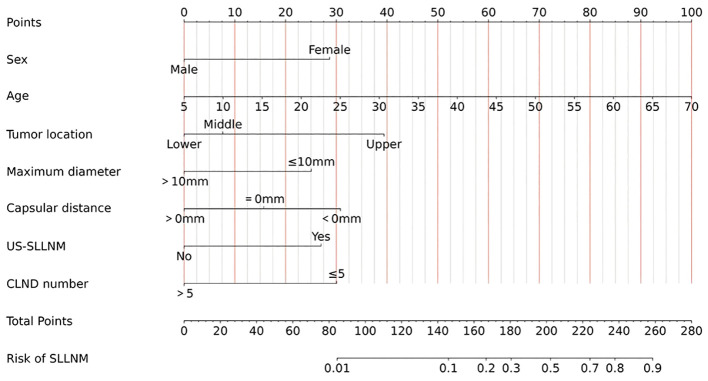
Nomogram of SLLNM in PTC patients.

Apart from this, static (**Figure 7**) and dynamic nomograms were constructed respectively: https://thyroidnomo.shinyapps.io/dynnomapp.

**Figure 8:**
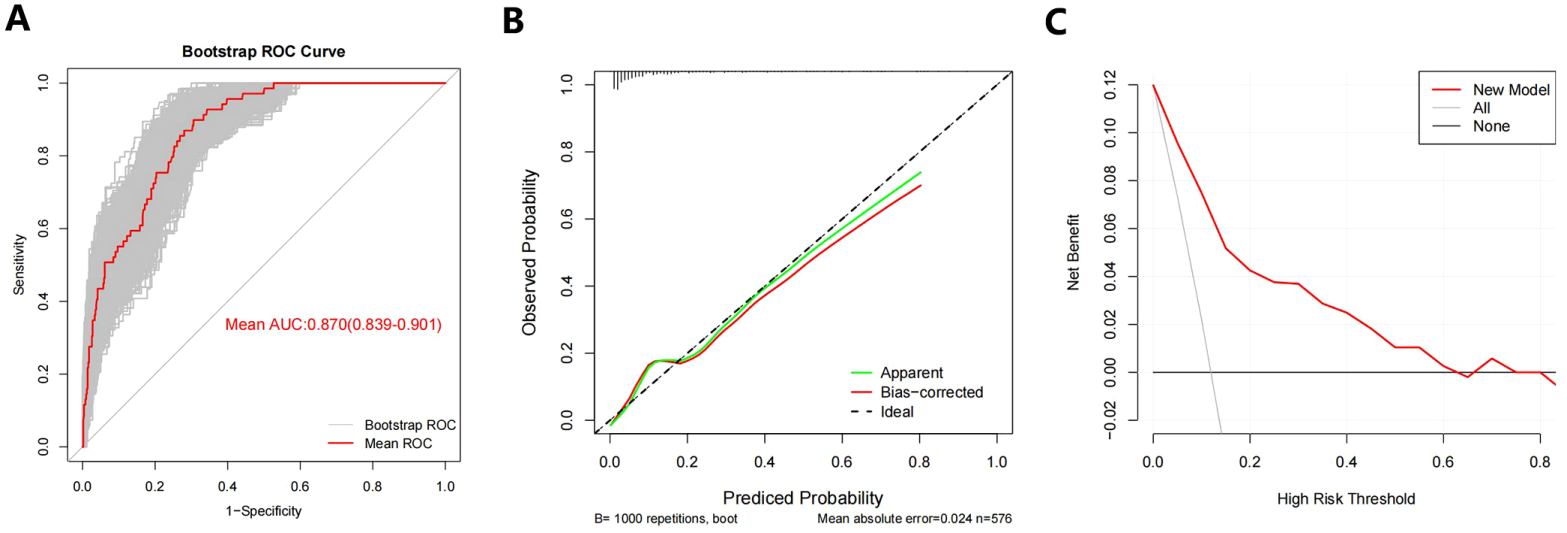
**(A)** Bootstrap ROC Curve; **(B)** Bootstrap Calibration Curve; **(C)** Bootstrap Decision curve analyses. ROC= receiver operating characteristic curve.

The new model had an AUC value of 0.869 (95% CI:0.833-0.906), with a sensitivity of 89.9% and a specificity of 69.4%. In order to evaluate the ability of our nomogram to predict SLLNM in PTC patients, we used 1,000 bootstrap resamplings for internal validation and obtained a mean AUC value of 0.870 (95% CI:0.839-0.901), showing good discrimination (**Figure 8A**); the calibration curve was also close to the ideal curve, with good consistency (**Figure 8B**). On the DCA curve, the net clinical benefit was seen from the 0 - 62% prediction probability interval (**Figure 8C**). According to the ROC curve, 7.9% is the optimal critical value of the model. When the prediction probability is 7.9%, a clinically significant net benefit can be obtained. Moreover, 89.9% (62/69) of PTC patients were correctly screened for SLLNM, which is of great significance in guiding the treatment of patients.

## 5 Discussion

In our external cohort, we evaluated the performance of three models for predicting SLLNM in PTC patients (Hu 2020 (6), Wang 2020 (7), and Zhao 2023 (10) nomograms). In the ROC curve analysis, the AUCs were all lower than the original models but also demonstrated good discrimination. However, when the Cutoff value at maximization of the Yoden index was applied to each of the three models, 29.0%, 36.2%, and 33.3% of SLLNM-positive patients, respectively, would be below the threshold, considered to be at low risk of SLLNM, and would not be considered for lymph node dissection of the lateral neck, which demonstrated a major limitation of the original models. As shown by the calibration curves (**Figure 4**), the original models fitted poorly overall in our external validation cohort. In the lower-risk patient cohort, the Hu 2020 nomogram (6) underestimated the actual risk to some extent, making it likely that SLLNM was present in patients expected to be at low risk. The Wang 2020 nomogram (7) was more consistent with the actual risk in the patient cohort with lower expected risk. In contrast, the Zhao 2023 nomogram (10) overestimated the actual risk, allowing more SLLNM-negative patients to receive intervention. The DCA curves for all three original models also show some net clinical benefit at the lower threshold probability intervals (**Figure 5**). These nomograms did not perform as well as the original cohort in our external cohort, probably because the baseline characteristics of the original cohort differed somewhat from our baseline characteristics. The difference in baseline characteristics may come from some subjective factors of tumor ultrasound characteristics and pathological characteristics, such as the judgment of tumor edge, ETE, etc. Furthermore, with advances in technology, fine-needle aspiration (FNA) of the lateral cervical lymph nodes is performed preoperatively to assess lymph node status. More precise clearance of metastatic lateral cervical lymph nodes has resulted in a higher incidence of LLNM in the study population, and there may be variations among centers in the timing and extent of FNA application, which may lead to bias in the selection of the populations in each cohort. In addition, surgeons’ subjective judgments of high risk for LLNM may be inconsistent across centers, which will also contribute to selection bias in the populations in each cohort.

To further refine the models, we took the approach of adding relevant risk factors by incorporating the results of Capsular distance, US-SLLNM, and CLND number into the original models, respectively (**Figure 3**). It can be seen that the performance of the models has been improved except for the Capsular distance which has no gain on the basis of Wang 2020.When all three risk factors were included in the three original models, the AUC increased significantly and exceeded that of the original cohort. As can be seen from the DCA curves in **Figure 5**, where the addition of the three factors significantly increased the range of threshold probabilities under the net benefit, as well as the percentage of net benefit under the same threshold probability. This means that these three factors can be included in the model to predict the outcome of SLLNM more accurately. Based on the results obtained above, we would like to explore further the relevant influencing factors involved in the above study by using a larger sample size to seek more stable independent predictors and construct a new model to identify PTC patients with SLLNM more accurately.

Expectedly, in our multivariate analysis, the three newly added variables, Capsular distance <0 mm, US-SLLNM, and CLND number ≤5 were all independent risk factors for SLLNM. The risk of SLLNM in patients with Capsular distance < 0mm was about 3.287 times higher than that in patients with Capsular distance > 0mm (OR 3.287, *P* = 0.019). In fact, this proves the study of Zhao 2023 that ETE under ultrasound is an independent risk factor for SLLNM (10). Furthermore, we could not precisely determine ETE under ultrasound and only assess it by envelope discontinuity. Therefore, we used a new variable, Capsular distance, to explore the effect on SLLNM. US-SLLNM incorporates the judgment of ultrasound on lymph nodes into the model. Even though ultrasound has low diagnostic efficacy for cervical lymph nodes, especially for the screening of central lymph nodes, ultrasound is routinely used as a preoperative examination in PTC patients, and it is still of certain value for the diagnosis of lymph nodes (28). The present ultrasound diagnosis of the SLLNM had a sensitivity of 59.4%, a specificity of 68.2%, and an accuracy of 67.2%. In this study, the CLND number was divided into cutoff values, and it was found that the risk of SLLNM in CLND ≤ 5 was approximately 2.244 times higher compared to CLND > 5 (OR 3.244, *P* < 0.001). This is consistent with the study of Zhu et al. (8), which also found that the reduction of CLND number was an independent risk factor for SLLNM, and the incidence of SLLNM was negatively correlated with CLND number (*P* < 0.05) (2, 10).

In addition, age, tumor location, and size were significantly associated with the risk of SLLNM, consistent with previous studies. The older the age, the higher the risk of SLLNM (6, 7, 14), but there is no consistency regarding the cutoff for age. Some studies have suggested 40 years of age (10, 25), while others have suggested 45 years of age as the cutoff for SLLNM (2, 29); Hu et al. and Dou et al. found that age >55 years was an independent risk factor for SLLNM by multivariate analysis (6, 15). Tumor size is generally defined as a cutoff point of 10 mm, and microcarcinomas ≤10 mm are considered more likely to develop SLLNM (2–5, 7, 8, 11, 13, 29). One study even suggested that SLLNM was more common when the primary tumor size ≤0.5 cm (OR = 12.9, *P* = 0.001) (12). This suggests that skip metastases may be in the early stages of cancer (25). However, Yang et al. (9) concluded that ≥10 mm was an independent risk factor for SLLNM, which may be because a larger proportion of SLLNM patients with larger tumors were located in the upper pole of the thyroid gland in that study, and the effects of tumor location and size were not distinguished, and the incidence of skip metastasis was only 3.4% in that study, which may have been a bias during the statistical analysis. Active surveillance has now become an alternative strategic choice for patients with micro PTC, requiring clinicians to carefully assess lateral cervical lymph node status. Numerous studies have shown a strong correlation with the development of SLLNM when the tumor is located in the upper pole (3–6, 8, 9, 14, 15, 17, 29–31). The same explanation is given: the upper pole of the thyroid lobe has a different lymphatic drainage system than the rest. Lymphatic drainage through the superior thyroid artery is transferred to the lateral lymph nodes (5, 7, 10, 32), and it is suggested that the lateral lymph nodes may be the first lymphatic drainage stations for tumors of the upper pole (15).

In this study, females became an independent risk factor for SLLNM (OR 2.740, 95% CI 1.279-5.870, *P*=0.009), which is in line with the studies of Zhao et al. and Jiwang et al. (2, 17), which also suggested that the female gender (OR 2.29, 95%CI 1.02-5.16) may contribute to an increased risk of SLLNM, again based on a larger sample. However, some studies concluded that gender was not significantly associated with SLLNM (3, 6–10), which may be due to inconsistencies in the inclusion criteria as well as grouping criteria for patients. In addition, the ratio of female to male patients in skip metastasis may also contribute to the heterogeneity between studies (3).

In summary, the model was also established to corroborate the findings of numerous studies and furthermore to summarize the reasons for the occurrence of SLLNM. It is more accepted that skip metastasis seems to develop by bypassing the normal lymphatic system of the central lymph nodes (5, 11, 16, 17) and is more commonly found in the lateral cervical zone III and zone II (4, 5, 11, 30). Using this model, it is possible to identify patients with PTC who are at high risk for SLLNM, for which we should carefully evaluate the lateral cervical lymph nodes and appropriately perform FNA of the lateral cervical lymph nodes or prophylactic lateral cervical lymph node dissection.

Our study is not without limitations. First, this study is a retrospective, single-center study, which may lead to selection and information bias. Second, in our external validation, we used approximate predictive probabilities because we did not obtain the regression parameters of the original models, and the validation results may have certain errors, but this is also a way to be close to the clinic. Further, the assessment of ultrasound characteristics involves some subjectivity, which means that observational differences between sonographers may be a factor affecting the results of external validation. In addition, the new variables we included and the reconstructed model need to be further validated in multi-center studies with larger samples.

## 6 Conclusion

We validated the predictive models of Hu 2020, Wang 2020, and Zhao 2023, and these nomograms showed good discrimination and some clinical benefit, but with varying degrees of underestimation or overestimation of the actual risk, and high false-negative rates. Besides, the added new variables all showed good gains in the original models, and the new dynamic nomogram was further constructed based on large samples, showing better performance.

## Data Availability

The original contributions presented in the study are included in the article/**Supplementary Material**. Further inquiries can be directed to the corresponding author.
