# Supplementary figures and images for: Single-center external validation and reconstruction of multiple predictive models for skip lateral lymph node metastasis in papillary thyroid carcinoma
Source: Front Endocrinol (Lausanne). 2024 Aug 23;15:1366679. doi: 10.3389/fendo.2024.1366679 (PMC11420524; doi:10.3389/fendo.2024.1366679)

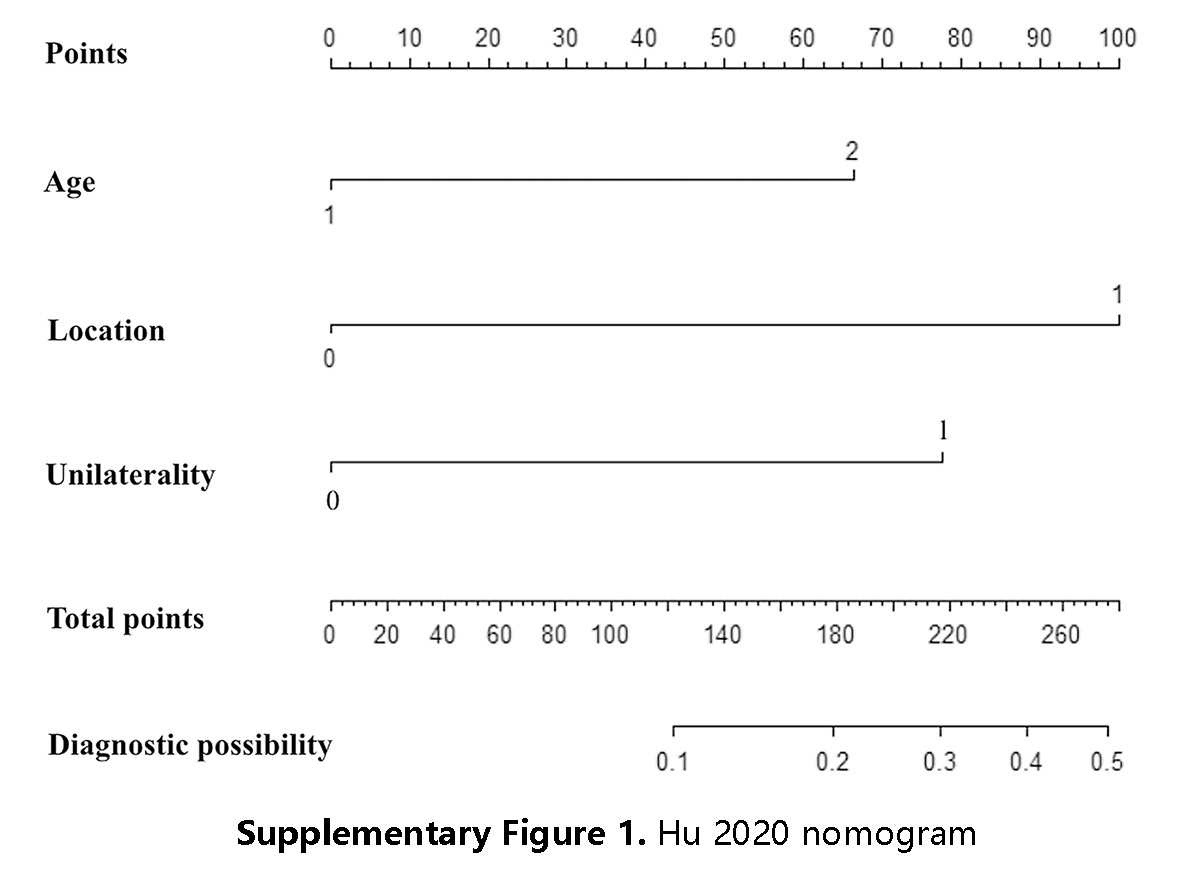

Supplement: Supplementary file 1 [file DataSheet1.zip › Supplementary_FIgures/Supplementary Figure 1.jpg]

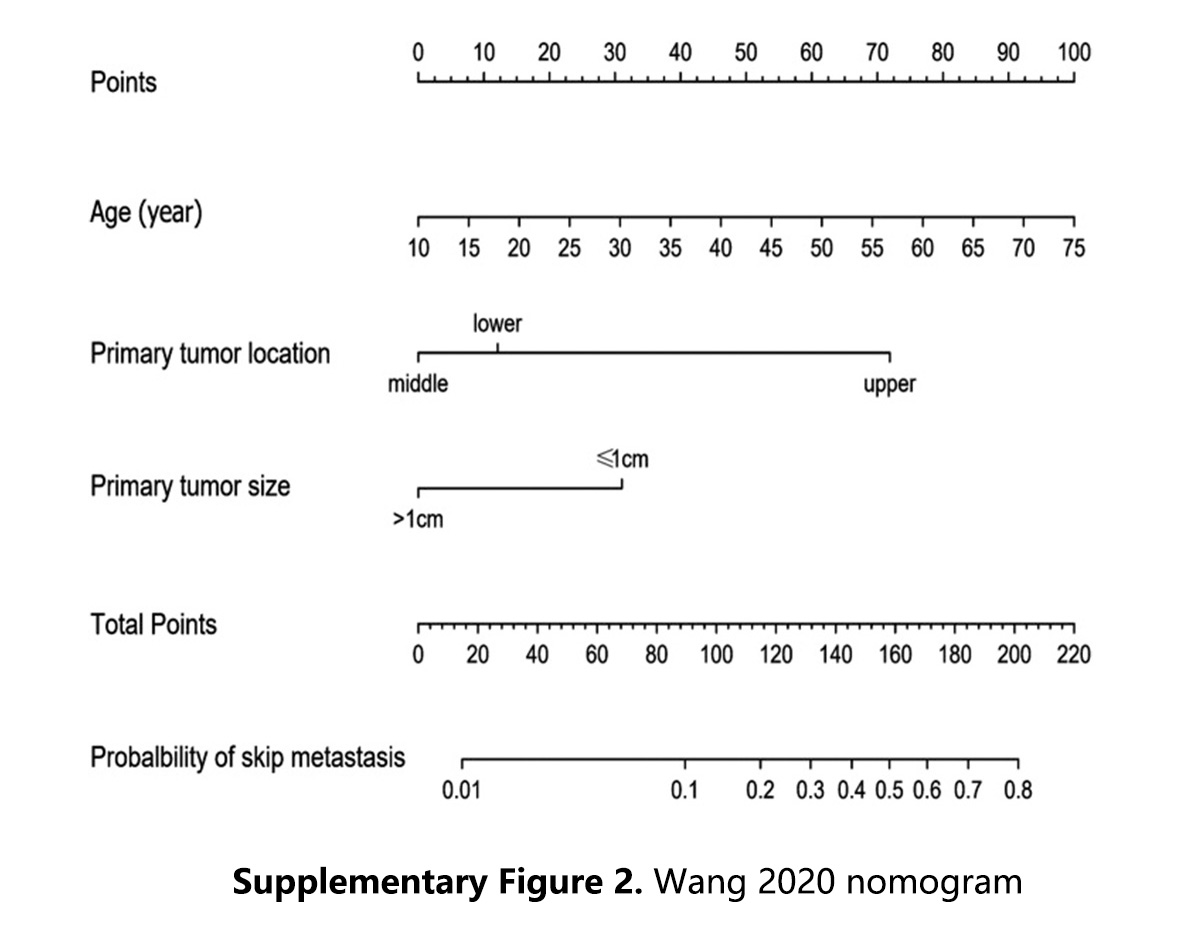

Supplement: Supplementary file 1 [file DataSheet1.zip › Supplementary_FIgures/Supplementary Figure 2.jpg]

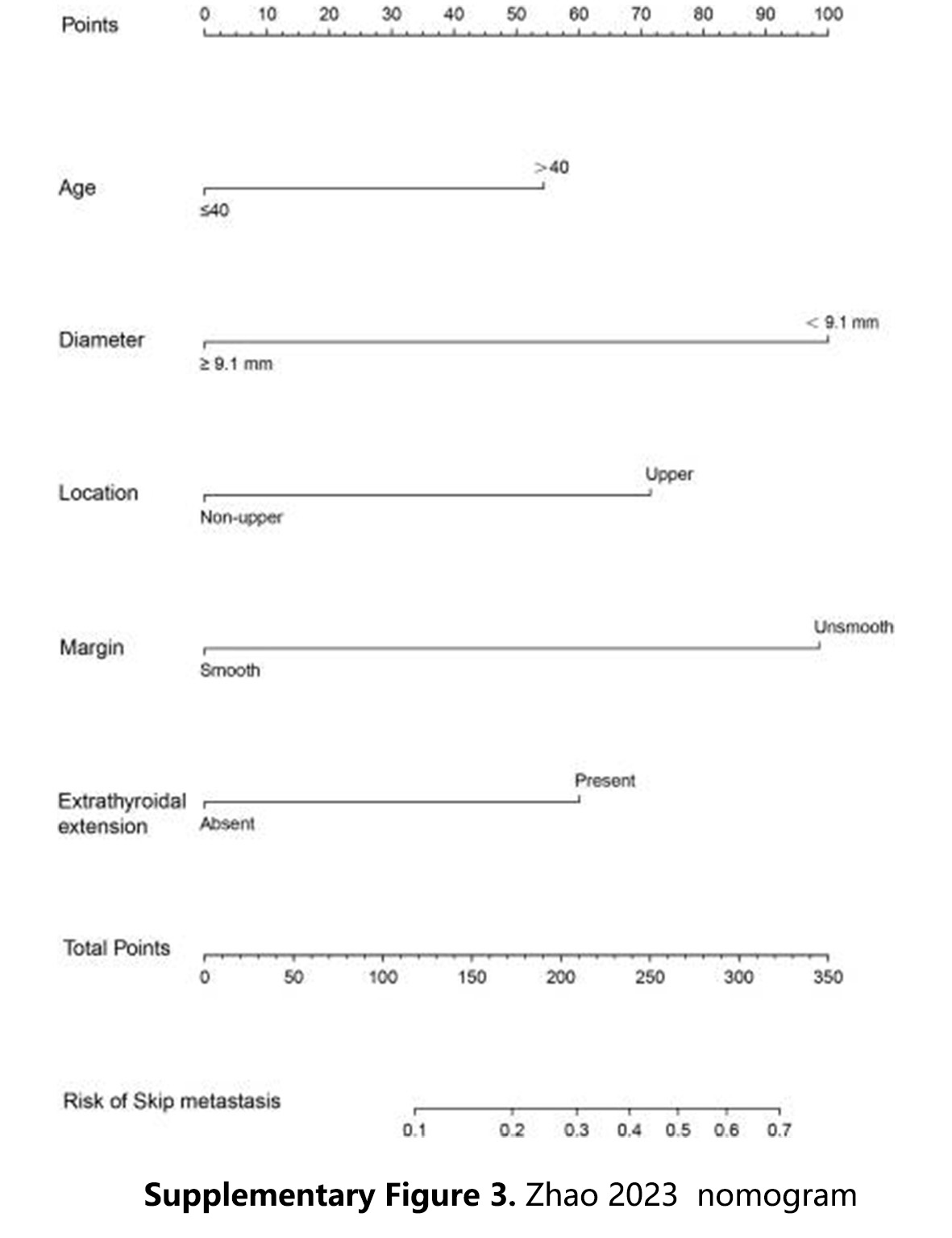

Supplement: Supplementary file 1 [file DataSheet1.zip › Supplementary_FIgures/Supplementary Figure 3.jpg]
