# Supplementary material for: Single-center external validation and reconstruction of multiple predictive models for skip lateral lymph node metastasis in papillary thyroid carcinoma
Source: Front Endocrinol (Lausanne). 2024 Aug 23;15:1366679. doi: 10.3389/fendo.2024.1366679 (PMC11420524; doi:10.3389/fendo.2024.1366679)
Supplement: Supplementary file 3 [file DataSheet3.docx]

Supplementary explanation

**1.Predictive probability of external validation**

The basic idea is to get the coordinates of the points in the graph through adobe illustrator (AI). Then calculate the corresponding scores for each variable through simple functions in excel.

**1.1**Get the score corresponding to each variable in AI

Build the panel in AI with the width set to 100mm for the length of the Points axis.
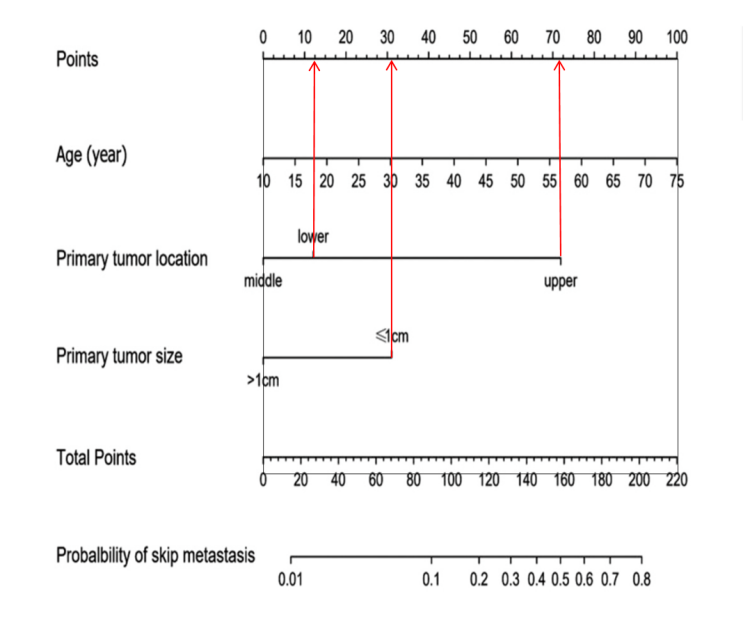


Categorical variables find the scores corresponding to each subvariable, and continuous variables find the scores corresponding to each unit ( for example, assuming that patient i's age is Age_i, the corresponding score is Age_i _score.It is known that the score is Age_min_score when age minimum = Age_min and Age_max_score when age maximum = Age_max; then there are:

Age_i_score = Age_min_score + (Age_i – Age_min)*(Age_max_score -Age_min_score) /(Age_max – Age_min)

**1.2**  Summarize the scores to arrive at Total Points.

**1.3** Calculating Predictive Probability from Total Points


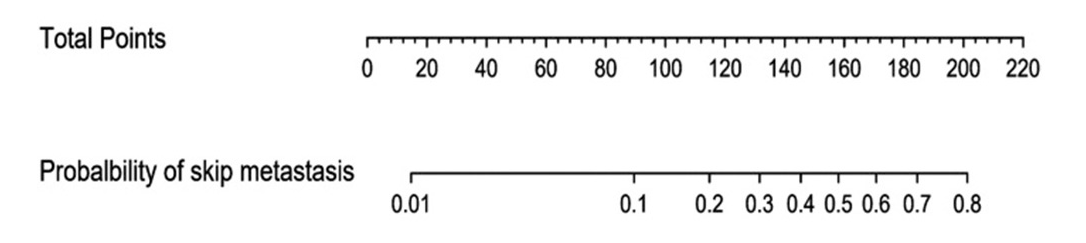


The general idea is that the change in probability between intervals is approximated as a linear change. That is, it is obvious that the intervals of the Total Points axis are fixed. The change in the values of the Predicted Probability axis is not linear, but within each interval, such as 0.01 - 0.1, 0.1 - 0.2, and so on, we consider the change to be linear. Outside of the two extremes, which were determined to be "<0.01" and ">0.9", the values of 0.01 and 0.9 had little effect on the overall evaluation of the model.

We use a similar method as in the first step to build a new drawing board in AI, this time with the width set to 220 mm for the length of the Total Point axis. After aligning the images, we obtain the Total Points values corresponding to each labeled value on the probability axis.

You can visit this web site to see how to do it:

<https://mp.weixin.qq.com/s/JYoIWG1mY9ApeoCUT2zdnQ>

After the above steps, **Excel3 (3.Predictive probability)** provides the results and related arithmetic functions for each indicator score, the total score, and the predicted probability.

The three nomograms for verification are shown in the S**upplementary Figures**.

**2、Operation code description**

The article is divided into two parts, one for external validation and the other for reconstructing the model. R (4.3.0) was used for all the production of the figures; SPSS (26.0) was used for the data description, and for the univariate and multivariate logistic regression analyses required for the reconstructed model. All the relevant code/script files (such as .R or .SPS) and data sheets have been uploaded.To run SPSS software, please load SPSS data and SPSS(sps); to run R software, load R data and R code. Both SPSS data and R data come from the same data table **(2.Analyze data)**.

A brief catalog of R and SPSS is listed below:


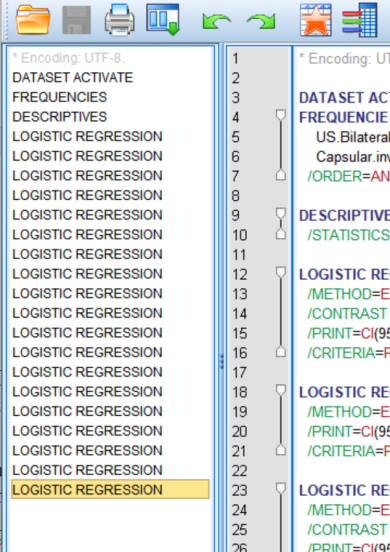

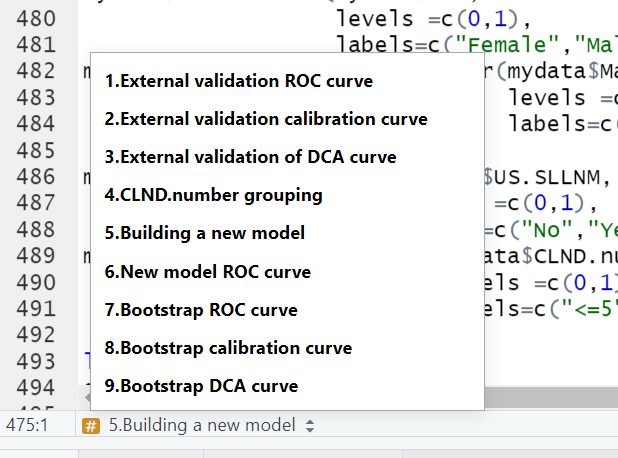
**2.1** Table 2 External cohort is the raw data we provided. SPSS (26.0) software was used to run SPSS (SPS) Rows 4-10 were used for data description.Data descriptions for Model 1,Model 2,Model 3 were obtained from tables in the respective literature.

**2.2** Table 3 and Table 4 are the summary forms of Figure 3, as shown in the R Code 1

**2.3** Table 5- Run SPSS (SPS) lines 12-117

**2.4** Table 6- Run SPSS (SPS) lines 119-130

**2.5** Figure 3- Run R Code 1

**2.6** Figure 4- Run R Code 2

**2.7** Figure 5- Run R Code 3

**2.8** Figure 6- Run R Code 4

**2.9** Figure 7- Run R Code 5 (including static and dynamic nomogram)

**2.10** Figure 8- Run R Code 6, 7, 8, 9

These are all the arithmetic instructions covered in this post, thank you for your patience and have a nice life!
